# Some residual malaria transmission may be “out of control” but “within reach” of current tools

**DOI:** 10.1073/pnas.2210568119

**Published:** 2022-09-12

**Authors:** Joseph Wagman, Christen Fornadel, Fredros Okumu

**Affiliations:** ^a^Malaria and Neglected Tropical Disease Program, PATH, Washington, DC 20001;; ^b^Innovative Vector Control Consortium, Liverpool L3 5QA, United Kingdom;; ^c^Environmental Health and Ecological Sciences Department, Ifakara Health Institute, Ifakara, United Republic of Tanzania;; ^d^School of Biodiversity, One Health and Veterinary Medicine, University of Glasgow, Glasgow, G61 1QH, United Kingdom

In their recent paper, Sangbakembi-Ngounou et al. elegantly show that many malaria mosquitos, including *Anopheles gambiae*, *Anopheles coluzzii*, and *Anopheles funestus* (collectively the most important vectors in Africa), frequently bite during the daytime and in outdoor, peridomestic spaces (1). The paper was exciting to read, and it is easy to imagine that 24- to 48-h mosquito surveillance windows will become more common, as will use of the circular statistics framework they are pioneering. This could provide more-complete, less-biased descriptions of malaria transmission than are achieved using traditional overnight collections.

By highlighting gaps in protection afforded by current vector control tools, notably insecticide treated nets (ITNs) and indoor residual spraying (IRS), they also make a compelling case that daytime and outdoor biting can contribute substantially to residual transmission—transmission that persists following the implementation of an effective malaria program (2). However, their results also emphasize the need to continue more nuanced discussions about the degree to which some gaps in protection might be specific to ITNs.

There is a common assumption that ITNs and IRS are redundant since they both involve the indoor use of insecticides, and that if a mosquito population is out of reach for one it is also out of reach for the other. As the authors point out, though, this might not always be the case, as “additional benefits of IRS come from the habit of some malaria vectors to rest inside dwellings, using them as refugia either before or after blood-feeding” (1). While dependent on the specific biology of the relevant vector(s), one important implication is that IRS can remain effective regardless of the time of blood-feeding: As long as vectors rest on a treated wall at some point, the actual timing of the blood meal is not critical. Neither is the specific location of biting: Evidence of an expanded reach for IRS is seen in some *An. funestus* and *Anopheles arabiensis* populations that readily bite humans outdoors, evading ITNs, yet still enter houses to rest and are controlled by effective IRS (3–5). Furthermore, the general mass-killing impact of effective indoor-insecticidal interventions can control even vectors that exhibit outdoor biting across Africa (6, 7).

Much residual transmission will likely be driven by exophagic, zoophilic “secondary” vectors less likely to enter houses at any point during their lifecycle (8, 9). We readily acknowledge that new approaches and tools to address these control gaps are needed (10). However, as reiterated by Sangbakembi-Ngounou et al. (1), the problem of residual malaria transmission is not that simple and often involves the “usual vector suspects” exhibiting opportunistic feeding and resting behaviors. While ITNs are most effective during nighttime sleeping hours, IRS is effective round-the-clock and can provide protection even in areas with daytime vector activity. Especially in communities that rely exclusively on ITNs, not all residual malaria transmission may be “out of control” with respect to IRS. Acknowledging budget constraints, it may be time to rethink single intervention approaches to achieving universal coverage and reconsider recommendations on combining IRS and ITNs.
